# Validation of a modified version of the Spanish Geriatric Oral Health Assessment Index (GOHAI-SP) for adults and elder people

**DOI:** 10.1186/s12903-020-1047-3

**Published:** 2020-02-19

**Authors:** Javiera Aguirre-Bustamante, Francisco Javier Barón-López, Francisco Jesús Carmona-González, Napoleón Pérez-Farinós, Julia Wärnberg

**Affiliations:** 1 Facultad de Odontología, Universidad San Sebastián, Lientur 1457, Concepción, 4080871 Chile; 2 Department of Nursing, School of Health Sciences, University of Málaga - Instituto de Investigación Biomédica de Málaga (IBIMA), Arquitecto Francisco Peñalosa, 3, Málaga, 29071 Spain; 3 Department of Public Health, School of Medicine, University of Málaga - Instituto de Investigación Biomédica de Málaga (IBIMA), Boulevard Louis Pasteur s/n, 29071 Málaga, Spain; 4 Unidad de Gestión Clínica Torrequebrada, Distrito de Atención Primaria Costa del Sol. Servicio Andaluz de Salud, Benalmádena, 29630 Málaga Spain; 5 Centro de Investigación Biomédica en Red Fisiopatología de la Obesidad y la Nutrición (CIBEROBN), Institute of Health Carlos III, Madrid, Spain

**Keywords:** GOHAI, Oral health-related quality of life, Gerodontology

## Abstract

**Background:**

The Geriatric Oral Health Assessment Index (GOHAI) was developed and validated in 1990 and translated into Spanish in 1999. Since then, the original version has been used in numerous studies, but it has not been re-evaluated in terms of language in the new generations of older adults. The purpose of this study is to confirm the validity of the Spanish version of the Geriatric Oral Health Assessment Index (GOHAI-SP) after three decades to be used as part of an ongoing field trial.

**Methods:**

The GOHAI-SP was pilot tested in a focus group to confirm linguistic comprehension. A version with minor language changes was administered to individuals with metabolic syndrome aged 55–75 years from one health care district in southern Spain as part of an ongoing field trial (PREDIMED-Plus). Clinical evaluation included assessment of dental and periodontal status. The psychometric properties of the GOHAI-SP were evaluated through stability and internal consistency measures, and concurrent and discriminant validity were assessed.

**Results:**

The new version of the GOHAI-SP was administered to 100 individuals. The application time was reduced by 7 min. The alpha value for reliability was 0.87. The item-scale correlation coefficients ranged from 0.54 to 0.75, and the test–re-test correlation for the total score was 0.75. There were inverse correlations between GOHAI-SP scores and the number of lost teeth and the decayed-missing-filled teeth index (*p* < 0.001).

**Conclusions:**

The GOHAI-SP questionnaire remains a valid and useful tool to assess oral health-related quality of life in primary health care settings. A linguistic update of the questionnaire brought improvements to the instrument application.

**Trial registration:**

The PREDIMED-Plus trial is registered in the ISRCTN registry with reference number ISRCTN89898870. Registration date: 4th July 2014.

## Background

The assessment of oral health-related quality of life (OHQoL) is essential for health promotion, disease prevention and efficient management of public health policies. In 1990, Atchinson and Dolan developed and tested the Geriatric Oral Health Assessment Index (GOHAI) [1], a self-reported questionnaire designed to assess oral health problems affecting quality of life (with 3 dimensions: 1. Physical function, including eating, speech and swallowing; 2. Psychosocial function, including worry or concern about oral health, self-image, self-consciousness about oral health, and avoidance of social contact because of oral problems. 3. Pain or discomfort) in North American elderly adults.

The questionnaire has been translated into several languages, and GOHAI versions are available in German [2], Chinese [3], Dutch [4], Spanish [5], French [6], Hindi [7], Persian [8], Malay [9], Swedish [10] and Turkish [11]. Since its development over three decades ago, the GOHAI has become one of the most widely used OHQoL questionnaires, and it has acquired the status of gold-standard questionnaire.

The translation and validation of the GOHAI into Spanish (GOHAI-SP) was conducted in 1999 with acceptable psychometric properties (Table 1). Since then, this translation has been used in several studies [12–17]. A key concern is that the authors of this translation and validation concluded that their translation could benefit from improved language [5]. Despite this clear advice, and although the index’s psychometric properties have been reviewed, it has been used for three decades as originally presented and no linguistic revision of the original version has been performed. In addition to the authors’ straight recommendation to improve language in 1999, other elements of linguistic expression may have changed since then.

**Table 1:** Spanish version of the geriatric oral health assessment index

Item	Question: In the past three months					
	Pregunta: ¿En los últimos tres meses	S	F	AV	RV	N
1	How often did you limit the kinds or amounts of food you eat because of problems with your teeth or dentures?					
	¿Cuántas veces ha tenido que comer menos o cambiar de comida por culpa de sus dientes o de su dentadura?	1	2	3	4	5
2	How often did you have trouble biting or chewing any kinds of food such as firm meat or apples?					
	¿Cuántas veces ha tenido problemas al masticar comidas como la carne o las manzanas?	1	2	3	4	5
3	How often were you able to swallow comfortably?					
	¿Cuántas veces ha tragado usted bien?	5	4	3	2	1
4	How often have your teeth or dentures prevented you from speaking the way you wanted?					
	¿Cuántas veces no ha podido usted hablar bien por culpa de sus dientes o dentadura?	1	2	3	4	5
5	How often were you able to eat anything without feeling discomfort?					
	¿Cuántas veces no ha podido comer las cosas que usted quería sin tener ninguna molestia?	1	2	3	4	5
6	How often did you limit contact with people because of the condition of your teeth or denture?					
	¿Cuántas veces no ha querido salir a la calle o hablar con la gente por culpa de sus dientes o dentadura?	1	2	3	4	5
7	How often were you pleased or happy with the appearance of your teeth and gums or dentures?					
	¿Cuándo usted se mira al espejo, cuántas veces ha estado contento de cómo se ven sus dientes o su dentadura?	5	4	3	2	1
8	How often did you use medication to relieve pain or discomfort around your mouth?					
	¿Cuántas veces ha tenido que utilizar algún medicamento para aliviar el dolor de sus dientes o las molestias en su boca?	1	2	3	4	5
9	How often were you worried or concerned about problems with your teeth, gums or dentures?					
	¿Cuántas veces ha estado preocupado o se ha dado cuenta de que sus dientes o su dentadura no están bien?	1	2	3	4	5
10	How often did you feel nervous or self-conscious because of problems with your teeth, gums or dentures?					
	¿Cuántas veces se ha puesto nervioso por los problemas de sus dientes o de su dentadura?	1	2	3	4	5
11	How often did you feel uncomfortable eating in front of people because of problems with your teeth or dentures?					
	¿Cuántas veces no ha comido a gusto delante de otras personas por culpa de sus dientes o dentadura?	1	2	3	4	5
12	How often were your teeth or gums sensitive to hot, cold or sweets?					
	¿Cuántas veces ha tenido molestias o dolor en sus dientes por el frío, el calor o las cosas dulces?	1	2	3	4	5

*S* Siempre/Always, *F* Frecuentemente/Frequently, *AV* Algunas veces/Sometimes, *RV* Rara vez/Seldom, *N* Nunca/Never. Items 3 and 7 have an inverse value relative to the rest of the items

It is possible that the new generation of adults has changed in different aspects. There is greater access to the use of technologies, such as cell phones and the internet. Older adults are better informed and have knowledge on disease treatment and prevention. Life expectancy and educational level have increased along with socio-economic conditions and employment [18].

Even a minor modification of a questionnaire maintaining its original structure requires re-verification of its psychometric characteristics [19, 20].

Considering that this questionnaire was developed more than three decades ago and translated into Spanish in 1999 and the characteristics of older adults may change over time, this study aimed to test the performance and comprehension of the Spanish translation of the GOHAI (GOHAI-SP) in a population of Spanish overweight adults aged 55–75. The specific aims of this study were to assess the comprehension of the GOHAI-SP by adults and elder people, to create a new modified version of the GOHAI-SP if necessary and to validate this new version.

## Methods

### Study subjects

A sample of well-characterized participants from an ongoing trial (PREDIMED-Plus) was used. PREDIMED-Plus is a multi-center randomized trial testing the effect of Mediterranean diet associated with physical exercise and behavioral therapy for primary prevention of cardiovascular disease in Spain. PREDIMED-Plus followed *CONSORT* guidelines for reporting [21]. Briefly, men aged 55 to 75 years and women aged 60 to 75 years who presented body mass index (BMI) ≥27 kg/m^2^ and < 40 kg/m^2^, no cardiovascular disease and at least 3 metabolic syndrome components [22] were eligible to participate in the study [23]. The exclusion criteria included illiteracy, inability to provide written informed consent, previous history of cardiovascular disease, any chronic medical condition (cancer, inflammatory bowel disease, cirrhosis, etc.), acute infectious processes, institutionalization, psychiatric disorders, any condition hindering regular physical activity, alcohol and drug abuse, use of specific medications (cytotoxic agents, immune suppressors, etc.) and important weight loss within a short time period.

All participants provided written informed consent to undergo an interview and clinical examination at their assigned primary health care center.

A sample of 20 volunteers from PREDIMED-Plus study (10 men and 10 women) aged 55–75 years from Torrequebrada Health Care Center (Benalmádena, Málaga, Spain) was selected for an initial focus study to assess comprehension of the current GOHAI-SP version. Twenty other volunteers from PREDIMED-Plus study (other 10 men and 10 women) aged 55–75 years from Torrequebrada Health Care Center (Benalmádena, Málaga, Spain) were selected for a second focus study to be performed after modifications to the GOHAI-SP. In addition, a sample of participants was selected for validation of the modified GOHAI-SP. This sample size was calculated to ensure a reliable estimation of Cronbach’s alpha, considering that the test has 12 items, an expected alpha of 0.85, and a 95% CI lower bound greater than 0.81. Thus, a sample size of *n* = 96 was obtained, which was rounded to 100.

### The GOHAI questionnaire

The GOHAI developed in 1990 by Atchison and Dolan [1] consists of 12 items with answers on a Likert scale. The questionnaire involves 3 dimensions (1. Physical function, including eating, speech and swallowing; 2. Psychosocial function, including worry or concern about oral health, self-image, self-consciousness about oral health, and avoidance of social contact because of oral problems. 3. Pain or discomfort) related to daily-life situations associated with the oral cavity in the last 3 months. The sum of the 12 items creates an “additive score” (ADD-GOHAI). Each item is scored from 1 to 5 points; therefore, the total points range from 12 to 60. In the absence of established population standards, an ADD-GOHAI score of 57–60 was considered high, 51–56 moderate, and less than 50 low. Thus, a higher score indicates better oral health and quality of life than a lower score.

The “simple count score” (SC-GOHAI) is obtained by adding one point every time an item is answered with “sometimes”, “often” or “always”. Therefore, the SC-GOHAI ranges from 0 to 12. A higher score indicates poorer oral health than a lower score. The 1999 Spanish translation was used in this study.

### Assessment of the GOHAI-SP: focus group studies

A focus group study was conducted with 20 volunteers from PREDIMED-Plus study (10 men and 10 women) aged 55–75 from Torrequebrada Health Care Center (Benalmádena, Málaga, Spain). The aim was to assess the comprehension and to test the performance of the GOHAI-SP in this population. The moderator selected for the focus study was a dentist. A group session (men and women together) was conducted at their primary health care center (Torrequebrada Health Care Center, Benalmádena, Spain). All participants received the GOHAI-SP for self-completion. The moderator encouraged the participants to talk and interact with each other about every question. Each of the participants gave his or her opinion about the comprehensibility of each of the 12 questionnaire items and the moderator registered it without making comments. In the end, the moderator provided his personal opinion related to the discussion. The total duration of the activity was 60 min.

### Modification of the GOHAI-SP

A group of experts including a dentist, a nurse, a statistician and a medical doctor analyzed and discussed the information collected from the focus study. The experts proposed modifications based on the conclusions of the focus group.

Two bilingual professional translators, a native Spanish speaker and a native English speaker, independently translated the changes made to the GOHAI-SP. The two translations obtained were compared and analyzed in a unique draft, which was translated into English by a professional native English translator. A modified version of the Spanish GOHAI (GOHAI-SPM) was then obtained. There were no changes in the meaning of the items. Table 2 shows the final version.

**Table 2:** Modified version of the spanish geriatric oral health assessment index

Item	Question: In the past three months					
	Pregunta: ¿En los últimos tres meses	S	F	AV	RV	N
1	How often did you limit the kinds or amounts of food you eat because of problems with your teeth or dentures?					
	¿Con qué frecuencia limita el tipo o cantidad de alimento que consume por problemas con sus dientes o prótesis?	1	2	3	4	5
2	How often did you have trouble biting or chewing any kinds of food such as firm meat or apples?					
	¿Con que frecuencia tiene problemas para morder o masticar cualquier tipo de alimento o comida más dura como carne o manzana?	1	2	3	4	5
3	How often were you able to swallow comfortably?					
	¿Con que frecuencia es capaz de tragar de manera cómoda/confortable?	5	4	3	2	1
4	How often have your teeth or dentures prevented you from speaking the way you wanted?					
	¿Con que frecuencia tus dientes o prótesis te impiden hablar de la manera que tú quieres?	1	2	3	4	5
5	How often were you able to eat anything without feeling discomfort?					
	¿Con que frecuencia ha podido comer cualquier cosa sin sentir disconfort/incomodidad?	1	2	3	4	5
6	How often did you limit contact with people because of the condition of your teeth or denture?					
	¿Con que frecuencia ha limitado el contacto con personas a causa de la condición de sus dientes o prótesis?	1	2	3	4	5
7	How often were you pleased or happy with the appearance of your teeth and gums or dentures?					
	¿Con que frecuencia está satisfecho o contento con el aspecto de sus dientes, encías o prótesis?	5	4	3	2	1
8	How often did you use medication to relieve pain or discomfort around your mouth?					
	¿Con que frecuencia ha utilizado medicamentos para aliviar el dolor o molestias en su boca?	1	2	3	4	5
9	How often were you worried or concerned about problems with your teeth, gums or dentures?					
	¿Con que frecuencia te preocupa/interesa los problemas de sus dientes, encías o prótesis?	1	2	3	4	5
10	How often did you feel nervous or self-conscious because of problems with your teeth, gums or dentures?					
	¿Con que frecuencia se siente nervioso o cohibido por problemas con sus dientes, encías o prótesis?	1	2	3	4	5
11	How often did you feel uncomfortable eating in front of people because of problems with your teeth or dentures?					
	¿Con que frecuencia te has sentido incomodo comiendo frente a personas por problemas en tus dientes o prótesis?	1	2	3	4	5
12	How often were your teeth or gums sensitive to hot, cold or sweets?					
	¿Con que frecuencia tus dientes han estado sensibles al calor, frío o dulce?	1	2	3	4	5

*S* Siempre/Always, *F* Frecuentemente/Frequently, *AV* Algunas veces/Sometimes, *RV* Rara vez/Seldom, *N* Nunca/Never. Items 3 and 7 have an inverse value relative to the rest of the items

A second focus study was carried out with 20 other volunteers from PREDIMED-Plus study (10 men and 10 women) aged 55–75 from Torrequebrada Health Care Center (Benalmádena, Málaga, Spain) prior to the validation study to evaluate the linguistic changes to the questionnaire (GOHAI-SPM). The participants answered the GOHAI-SPM questionnaire. The test-retest was performed with the same volunteers, leaving 1 week between both tests. The same methodology was used.

### Validation of the modified GOHAI-SP: Oral health assessment in a primary health care setting

A cross-sectional study was conducted between June 2015 and May 2016 with 100 participants aged 55–75 years from Torrequebrada Health Care Center (Benalmádena, Málaga, Spain), randomly selected from PREDIMED-Plus study database (not including the volunteers of the previous focus groups) with the aim to validate the GOHAI-SPM and to assess its psychometric properties.

During the interview, data on sociodemographic variables, self-perceived oral health (SPOH) and GOHAI-SPM scores were collected.

The protocol of this study was authorized by the Health Research Ethics Committee of Junta de Andalucía (Andalusian government). All participants provided informed consent prior to study participation.

#### Study protocol

All oral examinations were carried out in a dental box of Torrequebrada Primary Care Health Center. An oral exam was performed by a trained dentist using sterile gloves, a mask, a dental mirror, and a sterilized Hu-Friedy periodontal probe. The number of present, missing, decayed and filled teeth was assessed, and the presence of dental prostheses was recorded. The decayed-missing-filled teeth (DMFT) index was calculated [24]. Probing depth (PD) and clinical attachment level (CAL) were measured at six sites per tooth (mesio-buccal, mid-buccal, disto-buccal, mesio-lingual, mid-lingual, disto-lingual), excluding the third molars. Periodontitis was defined as the presence of four or more teeth with PD > 4 mm and CAL > 3 mm at one or more sites.

### Analysis

#### General psychometric properties

The psychometric properties of the GOHAI-SPM were evaluated. Cronbach’s alpha [25] was obtained to assess internal consistency and homogeneity among the items. Cluster analysis was performed to evaluate the questionnaire construct validity.

#### Validity

Concurrent validity of the questionnaire was determined by the degree to which the GOHAI-SP scores were related to the scores for self-perceived oral health and clinical variables (inter-item and item-scale correlations) and was assessed through Pearson’s correlation coefficient.

Convergent validity was determined by the association between GOHAI-SP scores and the objective assessment of dental and periodontal status. Linear regression models were used to estimate the effect of the dental and periodontal health variables. The dependent variables were ADD-GOHAI and SC-GOHAI, and the independent variables were self-perceived oral health, having more than 12 teeth [26], having a decayed tooth, and the presence or absence of periodontal disease. The models were adjusted for sociodemographic variables (gender, age, marital status, educational level and smoking status). Means and standard deviations of the dependent variables based on the groups defined by the independent variables were calculated and compared in the models.

To analyze the data, the Statistical Package for the Social Sciences (SPSS) software version 22 (IBM corporation, Armonk, New York, USA) was used. A *p*-value below 0.05 was considered statistically significant.

## Results

### Assessment of the GOHAI-SPM: focus group studies

The average initial response time of the GOHAI-SPM was 20 min.

Most participants (16 out of 20) stated that some expressions should be modified to improve their comprehension of the questionnaire, and they recommended different wording. Four participants did not object to the original wording but agreed that a minor change would make the questionnaire clearer. Eighteen of the 20 participants stated that the items should be converted into colloquial, straightforward Spanish so that it would be easily understood by most elder people in Spain.

### Modification of the GOHAI-SP

The group of experts decided to change the wording of the items while maintaining their original structure. They observed that some words had been incorrectly translated into Spanish. In the translation, the items that contained the words “How often” were modified. The phrase “Con qué frecuencia” (“How often”) was used in the GOHAI-SPM instead of “Cuántas veces” (“How many times”) because those items are related to the frequency with which an event occurs rather than the number of times it occurs (always, frequently, rarely or never). In addition, some terms were adapted to more commonly used words, such as “denture” instead of “prosthesis”.

### Assessment of the GOHAI-SPM

In the second focus study evaluating the linguistic changes to the questionnaire, there was a 7-min decrease in the response time. The participants understood the questions. There was no problem of understanding. Explanations by the administrator were not necessary for the participants to understand the questionnaire.

### Validation of the GOHAI-SPM: Oral health assessment in a primary health care setting

Oral health assessment was performed on a total of 100 participants. The average ADD-GOHAI and SC-GOHAI scores were 43.8 and 5.06, respectively. Table 3 shows the participants’ sociodemographic and medical characteristics.

**Table 3:** Sociodemographic, oral health and medical characteristics of the participants

	Mean (SD) or % (n)
Age (years)	66.0 (4.8)
Women	41.5 (39)
Married	79.3 (73)
Smokers	15.2 (14)
Educational level	
University	21.7 (20)
Secondary education	37.0 (34)
Primary education	41.3 (38)
Diagnosed with diabetes	45.7 (42)
Diagnosed with hypertension	43.9 (43)
ADD-GOHAI (score)	43.8 (9.9)
SC-GOHAI (score)	5.1 (3)
Self-perceived oral health	
Excellent-very good	7.4 (7)
Good	42.6 (40)
Enough-Poor	50.0 (47)
Frequency of tooth brushing	
Once a day	34.0 (32)
Twice or more times a day	66.0 (62)
Frequency of visits to the dentist	
Irregular	34.0 (32)
Once or twice a year	65.9 (62)
Present teeth (n)	18 (9.2)
Missing teeth (n)	10 (9.2)
Wearing a complete or partial removable denture	39.4 (43)
Diagnosed with periodontal disease	59.6 (56)
Probing depth (mm)	2.36
Attachment level loss (mm)	2.49

*SD* standard deviation, *ADD-GOHAI* additive score of GOHAI, *SC-GOHAI* simple count score of GOHAI, *GOHAI* General Oral Health Assessment Index

The frequencies and distributions of the 12 GOHAI-SPM items are shown in Table 4.

**Table 4:** Frequency (%) of answers to the 12 items of the GOHAI-SPM

Item	Always-Frequently	Sometimes- Seldom	Never
How often did you limit the kinds or amounts of food you eat because of problems with your teeth or dentures?	30.8	26.6	42.6
How often did you have trouble biting or chewing any kinds of food such as firm meat or apples?	42.5	18.1	39.4
How often were you able to swallow comfortably?	19.1	30.9	50.0
How often have your teeth or dentures prevented you from speaking the way you wanted?	13.8	9.6	76.6
How often were you able to eat anything without feeling discomfort?	35.1	29.8	35.1
How often did you limit contact with people because of the condition of your teeth or dentures?	10.6	14.9	74.5
How often were you pleased or happy with the appearance of your teeth and gums or dentures?	38.3	31.9	29.8
How often did you use medication to relieve pain or discomfort around your mouth?	10.6	36.2	53.2
How often were you worried or concerned about problems with your teeth, gums or dentures?	44.7	28.7	26.6
How often did you feel nervous or self-conscious because of problems with your teeth, gums or dentures?	10.6	35.1	54.3
How often did you feel uncomfortable eating in front of people because of problems with your teeth or dentures?	14.9	17.0	68.1
How often were your teeth or gums sensitive to hot, cold or sweets?	13.8	59.6	26.6

Most participants complained about difficulties chewing or biting (42.5%) and had concerns about their oral health (44.7%), although this difference was not statistically significant. Most participants did not report having any difficulty talking (76.6%).

Regarding psychological aspects, most participants were not concerned about having contact with others regardless of the state of their teeth (74.5%) and did not express uneasiness eating in front of other people (68.1%).

Table 5 shows the GOHAI-SPM inter-item and item-scale correlations.

**Table 5:** Inter-item and item-scale correlations for the GOHAI-SPM items

Item	1	2	3	4	5	6	7	8	9	10	11	12	Item-scale correlation
1	1												0.784^a^
2	0.609^a^	1											0.658^a^
3	0.510^a^	0.436^a^	1										0.612^a^
4	0.389^a^	0.338^a^	0.312^a^	1									0.591^a^
5	0.083^a^	−0.077^a^	−0.127^a^	0.086^a^	1								0.193^a^
6	0.481^a^	0.391^a^	0.536^a^	0.589^a^	0.012^a^	1							0.706^a^
7	0.393^a^	0.355^a^	0.221^a^	0.215^a^	0.044^a^	0.300^a^	1						0.568^a^
8	0.266^a^	0.175^a^	0.367^a^	0.217^a^	0.066^a^	0.186^a^	0.027^a^	1					0.423^a^
9	0.334^a^	0.209^a^	0.063^a^	0.079^a^	0.274^a^	0.188^a^	0.293^a^	0.094^a^	1				0.517^a^
10	0.548^a^	0.329^a^	0.368^a^	0.381^a^	−0.013^a^	0.507^a^	0.327^a^	0.253^a^	0.403^a^	1			0.708^a^
11	0.602^a^	0.487^a^	0.409^a^	0.532^a^	0.015	0.673^a^	0.359^a^	0.099^a^	0.307^a^	0.570^a^	1		0.757^a^
12	0.182^a^	0.073^a^	0.088^a^	−0.068^a^	0.097^a^	−0.015^a^	0.173^a^	0.141^a^	0.272^a^	0.263^a^	0.080^a^	1	0.299^a^

^a^Correlation is significant at the 0.05 level (two-sided)

Cronbach’s alpha for the GOHAI-SPM was 0.87, which shows high internal consistency and homogeneity among the GOHAI-SPM items. The item-scale correlation coefficients ranged from 0.29 to 0.78. When the 20 subjects of the second focus group retook the questionnaire 1 week after it was first administered, the test–re-test correlation for the total ADD-GOHAI score was 0.75. These values reflect the high questionnaire reliability.

The concurrent validity of the GOHAI-SPM scores and their association with SPOH status and the clinical variables is shown in Table 6.

**Table 6:** Association between GOHAI-SPM scores and self-perceived oral health status and clinical variables

Pearson’s correlation coefficients	ADD-GOHAI	SC-GOHAI
Self-perceived oral health	0.569^a^	−0.547^a^
Clinical measures		
Missing teeth	−0.509^a^	0.486^a^
Filled teeth	0.261^a^	−0.269^a^
DMFT index	−0.352^a^	0.326^a^

*ADD-GOHAI* additive score of GOHAI, *SC-GOHAI* simple count score of GOHAI, *DMFT* decayed-missing-filled teeth, ^a^Correlation is significant at the 0.05 level

High ADD-GOHAI scores were significantly correlated with a positive self-perception of oral health and a lower number of missing teeth and DMFT index. In the construct validity analysis (Table 7), confounding variables were considered.

**Table 7:** Estimated association of the GOHAI-SPM with SPOH and clinical and sociodemographic variables

			ADD-GOHAI		SC-GOHAI	
		Mean + SD	Multivariate coefficient	*p*-value	Multivariate coefficient	*p*-value
Sex	Man	45.6 ± 9.4				
	Woman	40.9 ± 9.8	−5.68 (−9.05 to −2.32)	*p* = 0.001*	2.40 (1.37 to 3.44)	*p* < 0.001*
Age	55 to 75 years	43.6 ± 9.8	0.15 (−0.15 to 0.45)	*p* = 0.313	−0.04 (− 0.13 to 0.05)	*p* = 0.422
Marital status	Single	36.8 ± 10.9				
	Married	44.8 ± 9.2	2.83 (−2.78 to 8.44)	*p* = 0.319	−0.65 (−2.37 to 1.08)	*p* = 0.457
	Divorced	41.9 ± 10.8	6.67 (0.30 to 13.04)	*p* = 0.040*	−2.25 (−4.20 to −0.29)	*p* = 0.025*
Educational level	University	46.3 ± 11.4				
	Secondary education	45.1 ± 8.2	−3.51 (−7.35 to 0.34)	*p* = 0.073	1.40 (0.21 to 2.58)	*p* = 0.021
	Primary education	41.2 ± 10.0	−6.89 (−10.65 to −3.14)	*p* < 0.001*	2.37 (1.21 to 3.53)	*p* < 0.001*
Smoker	Yes	38.4 ± 12.5				
	Ex-smoker 0–1 year	43.0 ± 7.2	−0.60 (−9.00 to 7.80)	*p* = 0.887	1.47 (− 1.11 to 4.05)	*p* = 0.261
	Ex-smoker 1–5 years	47.0 ± 13.0	7.49 (−1.40 to 16.38)	*p* = 0.097	−2.63 (−5.36 to 0.10)	*p* = 0.059
	Ex-smoker > 5 years	44.8 ± 8.6	4.91 (0.27 to 9.56)	*p* = 0.038*	−1.37 (−2.80 to 0.06)	*p* = 0.060
	Never smoked	44.3 ± 9.6	4.82 (−0.07 to 9.70)	*p* = 0.053	−1.38 (−2.88 to 0.12)	*p* = 0.071
Self-perceived oral health	Excellent-Very good	56.1 ± 3.3				
	Good	47.2 ± 7.8	−9.05 (−14.63 to −3.47)	*p* = 0.002*	2.68 (0.97 to 4.40)	*p* = 0.003*
	Enough-Poor	39.6 ± 8.4	−14.91 (−20.33 to −9.49)	*p* < 0.001*	4.75 (3.08 to 6.41)	*p* < 0.001*
Number of teeth	0–12 teeth	34.7 ± 10.1				
	13–32 teeth	46.2 ± 8.1	8.95 (4.68 to 13.23)	*p* < 0.001*	−2.83 (−4.14 to −1.51)	*p* < 0.001*
Periodontal disease	Yes	41.2 ± 10.8				
	No	46.4 ± 8.3	0.05 (−3.17 to 3.27)	*p* = 0.976	0.07 (−0.92 to 1.06)	*p* = 0.893

* *p* < 0.05 level, *SD* standard deviation, *ADD-GOHAI* additive score of GOHAI, *SC-GOHAI* simple count score of GOHAI, r-squared = 0.58, Adjusted r-squared = 0.5 (ADD-GOHAI); r-squared = 0.62, Adjusted r-squared = 0.55 (SC-GOHAI)

The participants with poorer oral health perception had lower scores of ADD-GOHAI than the participants with better perception. Participants with 13 or more teeth had better ADD-GOHAI scores than those with fewer than 12 teeth. The differences were significant.

The absence of periodontal disease was associated with higher ADD-GOHAI score, although the difference was not significant in the adjusted model.

Significant results were found for gender, marital status, educational level and smoking habits in the multivariate coefficient after the adjustment. Women and participants with only primary education had lower ADD-GOHAI scores than men and participants with university education, respectively. In contrast, participants who had never smoked and who were divorced scored significantly higher in the ADD-GOHAI.

## Discussion

Thirty years after its original creation, the GOHAI-SP was revisited, modified and tested in two focus groups. This study provided an opportunity to improve the instrument. Overall, some words from each item were modified to ensure proper understanding of the GOHAI-SP. Then, the questionnaire was administered to 100 participants with metabolic syndrome aged 55–75 years who lived in Benalmádena (Málaga, Spain) and were participating in PREDIMED-Plus trial. The results indicate that the modification of the GOHAI-SP questionnaires and the new version were successful. This comprehensible questionnaire was adapted to the common language used by elder Spanish people. After review, the index maintained satisfactory psychometric properties and appropriate internal consistency and reliability.

Although Locker’s model on which GOHAI is based [27] has been sometimes rebutted as a reliable portrayal of oral health, the GOHAI has been widely used to study oral health-related quality of life in different patients with different characteristics, and it has been available for use in different languages. In several clinical and epidemiological studies, it has been possible to consider multidimensional aspects of health and functional and psychosocial aspects.

Although the GOHAI questionnaire was validated decades ago, few studies have considered revisiting it before applying it to study a population. The psychometric properties of GOHAI-SP were evaluated in a Mexican population [14] and the results suggest that properties are acceptable. However, there were differences with the validation conducted in Spain explained by sociocultural differences (self-care oral health, missing teeth, chronic disease prevention and management, oral health services).

The colloquial language, specific cultural norms and references to the national health service can be modified. Therefore, it is necessary to study the psychometric characteristics of the measuring instrument before using it in a large-scale study. The assessment tools, culturally adapted and validated in different languages, can not only provide a comparison of the results obtained using the same tool but also be used for clinical practice to reduce operating costs in public health and to develop international studies in diverse cultures [28].

The participants of the second focus group stated that the questions of the GOHAI-SPM questionnaire were easier to understand. The items contained words from the local jargon and were adequate to get the answer they asked for. Additionally, the administration of the questionnaire was clearer, so that it could be completed in a shorter time and without the need for the dentist’s explanations.

The reliability of the Malayan version of the GOHAI [29] was assessed before use in a larger cohort of elder adults of different cultures. A Cronbach’s alpha of 0.67 was reached for the 12 GOHAI items. Similar results were obtained in the study of Sanchez-García et al. [14] in a Mexican population. The internal consistency analysis of the GOHAI-SP yielded a Cronbach’s alpha coefficient of 0.61 for the 12 items.

In our study, the Cronbach’s alpha coefficient of 0.87 is considered appropriate, with previous studies showing coefficients between 0.67 and 0.92. A modified version of the questionnaire has shown improved internal consistency. Our results reinforced this observation and they also revealed that the GOHAI-SPM can discriminate between oral health status and psychological and functional considerations. This provides valuable information about oral symptoms, and psychosocial and functional problems that cause discomfort or distress to the patient. Oral disorders in older adults compromised quality of life in the physical, psychological, social and environmental domains [30, 31]. This has been observed in previous studies involving other versions [9, 32–34].

Furthermore, the participants who reported poorer self-perceived oral health and had fewer teeth tended to have lower scores in the GOHAI-SP. This finding from this new version of the GOHAI-SP confirms the instrument validity for OHQoL evaluation and is similar to the results of other studies [4, 35–39].

The GOHAI-SPM cannot identify periodontal disease. However, aspects related with common periodontal conditions in the geriatric population should be considered in future studies to evaluate OHQoL in the elderly population. We consider that the questionnaire must be modified to include questions related to the impact of periodontal disease on elderly people’s quality of life, such as halitosis and/or tooth mobility, which are aspects that affect dental device functionality and personal relationships.

These aspects are not included in the GOHAI questionnaire, and there are few references in the literature to support this finding [32, 34–40].

Another concern about the GOHAI is that the combination of positive and negative items may make it difficult to be interpreted. However, we consider that our study shows that the Spanish version of GOHAI, and particularly this new version, partially gets around that difficulty.

The impact of certain diseases on general health can influence OHQoL [41–52]. Future research should also focus on developing tools for consistent OHQoL evaluation, taking into consideration other areas related to OHQoL to broaden the understanding of the concept of oral health, such as sociocultural environment, economic priorities, diet and systemic diseases [53].

There are other instruments for oral health assessment, such as OHIP, SOHSI, OIDP or the Oral Health Questionnaire [54]. Although we consider that the modified version of the Spanish GOHAI is valid for accurate assessment of oral health in adult and elder people, it is important to take into account those other instruments for particular situations where they can be useful.

## Conclusions

The modified version of the GOHAI-SP presents adequate psychometric properties and can be used as a useful tool for OHQoL evaluation in the elderly population. The use of this more modern version has advantages in the clinical application of the questionnaire. Updates to the questionnaire wording can reduce the application time and prevent the interviewer from needing to explain the items. This questionnaire can be applied for reference in future studies conducted by national and international researchers in Spanish-speaking communities with similar features.

## Data Availability

The datasets generated and analyzed during the current study are not publicly available due to national data regulations and ethical reasons, including the possibility that some information might compromise research participants’ consent because our participants only consented to the use of their data by the original team of investigators. However, these data are available from the corresponding author upon reasonable request by signing a data sharing agreement as approved by the relevant research ethics committees.

## Abbreviations

ADD-GOHAI: Additive score of GOHAI
CAL: Clinical attachment level
DMFT: Decayed-missing-filled teeth
GOHAI: Geriatric Oral Health Assessment Index
GOHAI-SP: Spanish version of the Geriatric Oral Health Assessment Index
GOHAI-SPM: Modified version of the Spanish Geriatric Oral Health Assessment Index
OHQoL: Oral health-related quality of life
PD: Probing depth
PREDIMED: Prevención con dieta mediterránea
SC-GOHAI: Simple count score of GOHAI
